# Real-time application of the Rat Grimace Scale as a welfare refinement in laboratory rats

**DOI:** 10.1038/srep31667

**Published:** 2016-08-17

**Authors:** Vivian Leung, Emily Zhang, Daniel SJ Pang

**Affiliations:** 1 Veterinary Clinical and Diagnostic Sciences, Faculty of Veterinary Medicine, University of Calgary, AB, T2N 4Z6 Canada; 2 Hotchkiss Brain Institute, University of Calgary, AB, T2N 4Z6 Canada

## Abstract

Rodent grimace scales have been recently validated for pain assessment, allowing evaluation of facial expressions associated with pain. The standard scoring method is retrospective, limiting its application beyond pain research. This study aimed to assess if real-time application of the Rat Grimace Scale (RGS) could reliably and accurately assess pain in rats when compared to the standard method. Thirty-two male and female Sprague-Dawley rats were block randomized into three treatment groups: buprenorphine (0.03 mg/kg, subcutaneously), multimodal analgesia (buprenorphine [0.03 mg/kg] and meloxicam [2 mg/kg], subcutaneously), or saline, followed by intra-plantar carrageenan. Real-time observations (interval and point) were compared to the standard RGS method using concurrent video-recordings. Real-time interval observations reflected the results from the standard RGS method by successfully discriminating between analgesia and saline treatments. Real-time point observations showed poor discrimination between treatments. Real-time observations showed minimal bias (<0.1) and acceptable limits of agreement. These results indicate that applying the RGS in real-time through an interval scoring method is feasible and effective, allowing refinement of laboratory rat welfare through rapid identification of pain and early intervention.

Pain in animals is commonly under-treated. This stems from numerous factors, including the limited availability of validated pain scales^1^,^2^,^3^,^4^. In laboratory rodents, analgesic administration rates as low as 15% have been reported for invasive procedures (e.g. orthopedic surgery, thoracotomy) and data variability related to the presence of pain and sporadic analgesic use is likely to act as a confounding factor during experimental studies^5^,^6^. Furthermore, some experimental designs allow analgesia to be withheld until established humane endpoints have been reached^5^. These endpoints, such as weight loss, are largely non-specific and little is known about their relationship to pain^7^. Early recognition of pain coupled with appropriate intervention would address these issues and support refinement of *in vivo* research^5^,^8^,^9^,^10^.

The recent development of rodent grimace scales has expanded our ability to assess pain in rodents^11^,^12^ and potentially addresses failures in translational pain research resulting from a reliance on evoked-response nociceptive testing^13^,^14^,^15^.

The Rat Grimace Scale (RGS) consists of four facial “action units” (orbital tightening, nose/cheek appearance, ear and whisker positions) which are scored using still images by an observer^12^. The RGS has been validated, showing content and construct validity and reliability (inter- and intra-observer)^12^,^16^. An analgesia intervention threshold has been derived for the RGS and it has been used to highlight discrepancies between nociception and spontaneous ongoing pain^13^,^16^. The development of both the RGS and Mouse Grimace Scale (MGS) has allowed reappraisals of analgesic efficacy in these species^8^,^9^.

In their current form, the RGS and MGS show great potential as research tools in the study of pain. However, the standard method of generating pain scores requires multiple steps: high quality video-recording, automated or manual selection of several images per time point and scoring^12^,^16^. These steps are time and labour intensive and consequently inhibit wider application of the scales. Performing real-time scoring with the RGS and MGS would broaden their applications, facilitating improvements in welfare through rapid, early and accurate identification of pain, thus bridging the gap from research tool to improving rodent care and welfare.

Real-time scoring has been attempted in mice^17^ and has been proposed, but remains untested, in rats^16^. Potential obstacles to real-time scoring are: 1. a change in behaviour in the presence of an observer (observer effect), 2. an inherent bias from the observer being able to observe the whole animal rather than just the head, as performed in the validation studies (observer bias) and 3. limited accuracy of real-time scoring of moving animals without the control offered by video playback.

We hypothesised that the standard video-based application of the Rat Grimace Scale could be successfully translated to real-time assessment. This hypothesis was tested through two specific aims: 1) assessing if results from two different real-time scoring methods are comparable to those collected through standard RGS methodology and 2) assessing the shortest observation period possible for real-time scores to remain comparable to standard RGS scores.

## Methods

### Ethical statement

All experiments were approved by the University of Calgary Health Sciences Animal Care Committee and performed in accordance with Canadian Council on Animal Care guidelines.

### Experimental animals

Forty-four male and female Sprague-Dawley rats (224–435 g) were obtained from the University of Calgary Animal Resource Centre surplus stock and Charles River, Canada. Animals were housed in pairs in polycarbonate or polysulfone rat cages (RC88D-UD, Alternate Design Mfg and Supply, Siloam Springs, Arizona, USA) with bedding of wood shavings, shredded paper, sizzle paper and a plastic tube for enrichment. The housing environment was controlled: light cycle of 12 hours on/12 hours off (lights on at 0700) and temperature and humidity settings of 23 °C and 22%, respectively. Laboratory rat pellets (Prolab 2500 Rodent 5P14, LabDiet, PMI Nutrition International, St Louis, MO, USA) and tap water were available ad libitum.

### Experimental procedures

All animals were habituated to the observer and observation chamber for three days. During these habituation sessions, each animal was placed in the observation chamber for approximately 10 minutes and handled by the observer for at least 20 minutes. Animals were offered a food reward (Honey Nut Cheerios™, General Mills, Inc., Golden Valley, Minnesota, USA) when handled. They were considered habituated when they voluntarily ate the food reward while being held by the observer.

**Figure 1:**
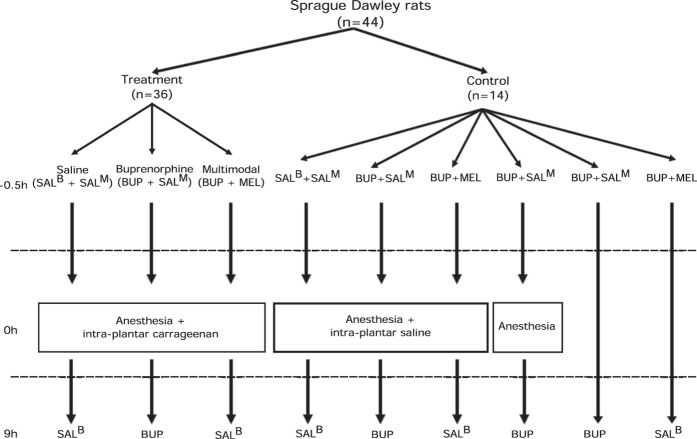
Flow chart depicting experimental pathway for each treatment group. SAL^B^, saline volume equivalent to buprenorphine dose. SAL^M^, saline volume equivalent to meloxicam dose. BUP, buprenorphine. MEL, meloxicam.

Sample sizes for treatment groups were chosen based on RGS data variability observed in previous publications^12^,^16^ with an alpha of 0.05, beta of 0.8 to detect a mean difference of 0.3. Injections were prepared by a third-party not involved in the experiment. All injections were performed between 0700 and 0915 hours and testing completed within the light period. Image scoring and real-time observations were performed by a single observer. Animals were block randomized into one of nine treatment groups (Fig. 1). Three treatment groups received intra-plantar carrageenan (100 microlitres of 1% λ-carrageenan dissolved in saline, Sigma-Aldrich, St. Louis, MO, USA) with either buprenorphine (0.03 mg/kg SC, Vetergesic, Champion Alstoe, Whitby, ON, Canada, n = 12), buprenorphine (0.03 mg/kg SC) and meloxicam (“multimodal analgesia group”, 2 mg/kg SC, Metacam 0.5% injection, Boehringer Ingelheim, Burlington, ON, Canada, n = 12), or saline (n = 12). A cross-over design was used for the control groups, with each animal receiving three control treatments with a minimum 10-day washout period between treatments (Fig. 1).

All animals received two sets of injections. The first was given 30 minutes before intra-plantar injection and the second 9 hours after intra-plantar injection (or equivalent time for the control groups). Injections at 9 hours were given after pain assessments were completed.

Intra-plantar injections were performed under brief general anaesthesia. Animals were placed individually in a plexiglass induction chamber and 5% isoflurane carried in oxygen (1 L/min) administered until loss of righting reflex occurred, at which point the animal was transferred to an adjacent counter (anaesthesia maintained by nose cone with 2% isoflurane in 1 L/minute oxygen) and placed in sternal recumbency on a heat pad. The left hind paw was extended caudally and the plantar surface wiped with 70% ethanol. The assigned treatment (carrageenan or saline) was injected subcutaneously into the plantar surface. Animals were then allowed to recover with 1 L/minute oxygen and returned to their home cages once the righting reflex had returned.

### Observations

Two video cameras (Panasonic HC-V720P/PC, Panasonic Canada Inc., Mississauga, ON, Canada) were placed at opposite ends of the observation chamber (28 × 15 × 21 cm). During real-time observation the observer was positioned perpendicular to the camera, and was free to move around without entering the cameras’ field of view. Three observation periods (V1, O+V, V2) were video-recorded consecutively. V1: video-recording was performed with no observer present. O+V: real-time observations were performed concurrently with video recording. V2: video-recording was performed with no observer present. Each observation period was 10-minutes long. Observations were performed at baseline (day before procedure) and 3, 6, 9 and 24 h after intra-plantar injections (or equivalent time for control groups).

### Image RGS scoring

Image scores (IMG) were generated as previously described, by selecting the best image from each consecutive 3-minute period of a 10-minute video^12^. Videos were relabelled by a third party not involved in image grabbing or scoring, blinding the observer to the rat, treatment and time point. The preferred image was a frontal view that clearly showed all action units. A profile view was selected if no frontal image of sufficient quality was available. Images were put into a presentation software (Microsoft PowerPoint, version 15.0, Microsoft Corporation, Redmond, WA, USA) and the slide order randomised before scoring. An average score was calculated from the three images from each video.

### Real-time RGS scoring

**Figure 2:**
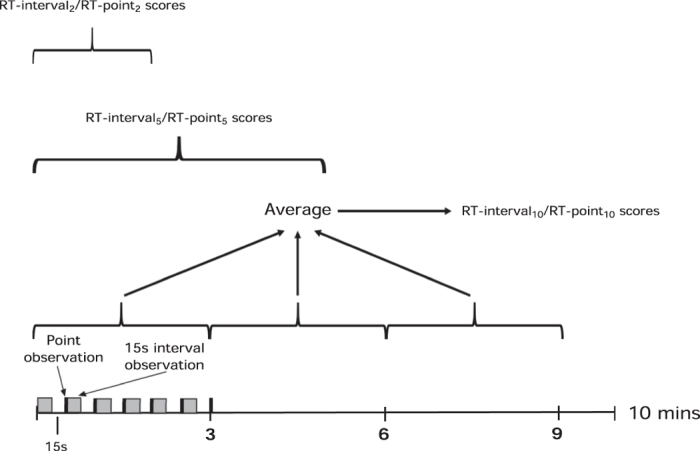
Cartoon of real-time observation methods. Observations alternate between point and 15 s interval observations. After a 15 s pause, the observations are repeated for the 10-minute observation period. Scores from each 3-minute block were averaged and 3 blocks averaged to give an overall score for the 10 minute period (real-time interval [RT-interval_10_] and real-time point [RT-point_10_]). Raw scores were also averaged over 5 (RT-interval_5_ and RT-point_5_) and 2 minutes (RT-interval_2_ and RT-point_2_).

Real-time (RT) scores were obtained using two methods: 1) a point observation alternating with 2) a 15 s interval observation, where the animal was observed for 15 s and assigned a single score for the period. Each method was repeated every 30 s for the 10-minute observation period, generating 18 scores of each type per animal. Similar to the standard method described for RGS scoring^12^, scores generated from both methods were averaged every three minutes to produce three separate scores and these averaged to yield a single score (RT-interval_10_ or -RT-point_10_). Real-time scores were also averaged from the first five and two minutes of the observation period (RT-interval_5_, RT-point_5_, RT-interval_2_, RT-point_2_) to compare shorter observation periods (Fig. 2).

Additionally, five single real-time scores from each 10 minute observation period were randomly selected (single RT-interval and single RT-point) to evaluate variability associated with single observations.

Real-time scoring and image grabbing was not performed if a rat was rearing (two paws raised off the chamber floor), sniffing, grooming or sleeping.

### Pica

A petri dish (given to each cage at the beginning of habituation period) was weighed at baseline and after the experiment as pica is a potential side effect of buprenorphine^18^. Pica was confirmed if there was evidence of petri dish fragments at necropsy examination (visual inspection of the stomach contents) or a decrease in the mass of petri dishes (>0.1 g) was observed.

### Statistical methods

Data analyses were performed using commercial software (Prism 6.07, GraphPad Software, La Jolla, CA, USA). Open source software (R 3.3.0, ‘MethComp’ package ver. 1.22.2) was used for the Bland and Altman method. Data were assessed for normality with a D’Agostino-Pearson omnibus normality test and parametric tests applied where data approximated a normal distribution. Repeated measures two-way ANOVA was used for between group comparisons with *post-hoc* tests if a significant main effect was observed: RT-interval and RT-point *versus* IMG scores (*post-hoc* Dunnett’s test), treatment groups (saline vs buprenorphine vs multimodal; *post-hoc* Tukey’s test), single RT-interval and single RT-point *versus* IMG scores (*post-hoc* Dunnett’s test), observer effect (RGS scores during observation periods with and without the observer present; *post-hoc* Tukey’s test). When it was not possible to obtain an RGS score for a rat at a given time point, an average of the scores obtained from other rats at the same time point was substituted to allow analysis. The Bland and Altman method for repeated measures was used to assess agreement between IMG scores and RT-interval or RT-point scores^19^. Control data were analysed with Friedman’s test with a *post-hoc* Dunn’s test. Differences were considered statistically significant if the computed two-tailed p value was less than 0.05. When available, p values are reported with 95% confidence intervals (95% CI). Data are presented as mean ±SD or median ±interquartile range. Graphs are plotted as mean ± SEM.

## Results

Four animals were excluded as a result of misinjection (carrageenan and buprenorphine group, n = 1; carrageenan and buprenorphine and meloxicam group, n = 1, carrageenan and saline group, n = 2), leaving 41 animals included in the final analysis. As the frequency of observations decreased, more missing observations occurred: 2 minutes (interval and point); 21/310 observations (missing/ total observations), 5 minutes; 8/310 observations, 10 minutes; 6/310 observations.

### Multiple interval and point observation scoring methods

**Figure 3:**
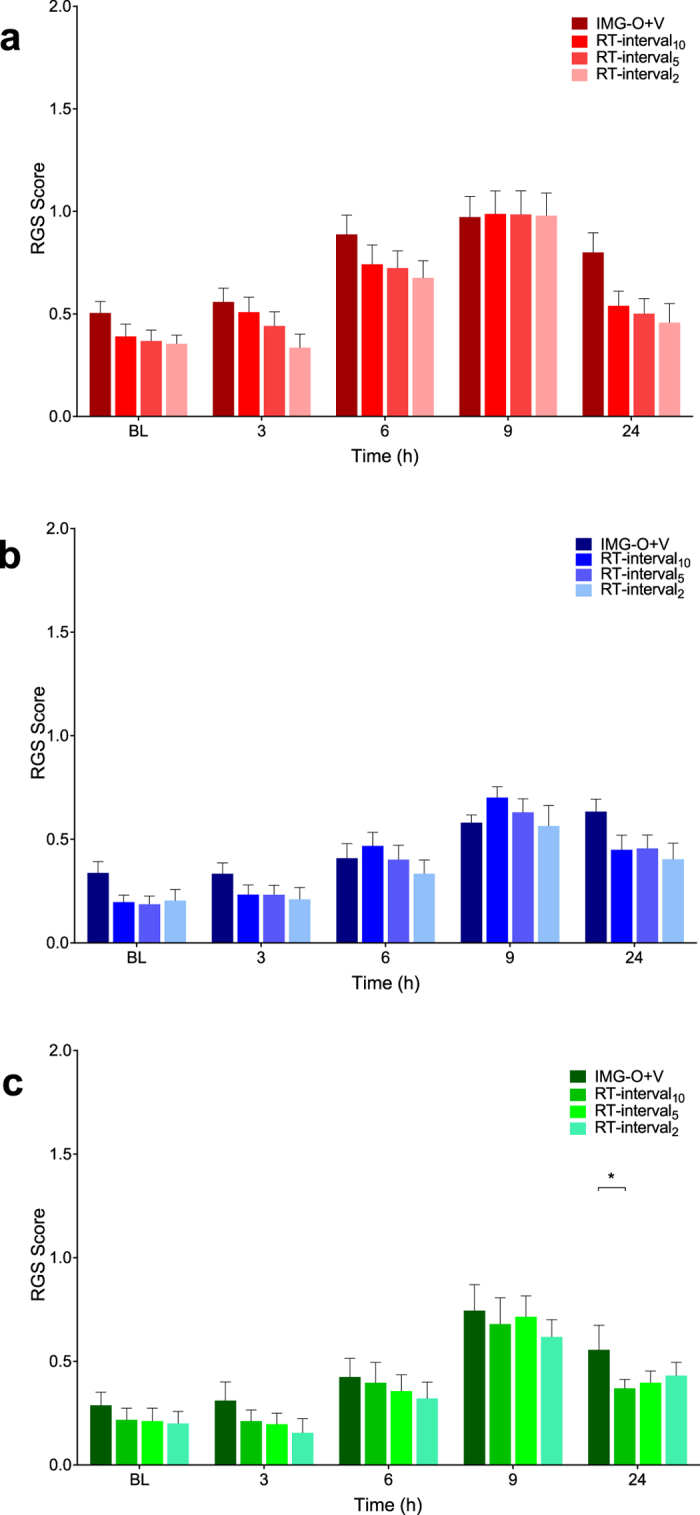
Real-time interval Rat Grimace Scale (RGS) scoring methods were comparable to standard RGS scoring. Saline (**A**) and buprenorphine (**B**): scoring methods had no significant effect on RGS scores (saline: p = 0.14; buprenorphine: p = 0.28). (**C**) scoring method was found to have an effect in the multimodal group. However, the difference was limited to the 24 hour time point (between IMG-O + V and RT10, p = 0.02). RT-interval = real-time interval RGS scoring. IMG-O + V = standard (video-based) RGS scoring. Data are mean ± SEM.

Agreement between real-time interval observation scoring methods (RT-interval_10_, RT-interval_5_, RT-interval_2_) were comparable to the standard RGS method (IMG-O+V, Fig. 3). No significant differences were observed between these observation methods at each time point in the saline (F = 1.92, df 3, p = 0.14, Fig. 3a) and buprenorphine (F = 1.32, df 3, p = 0.28, Fig. 3b) groups. A single difference was observed in the multimodal (buprenorphine and meloxicam) treatment group (F = 13.74, df 3, p < 0.0001) at the 24 hour time point between IMG O+V and RT-interval_10_ (p = 0.02, 95% CI: 0.02 to 0.35, Fig. 3c).

**Figure 4:**
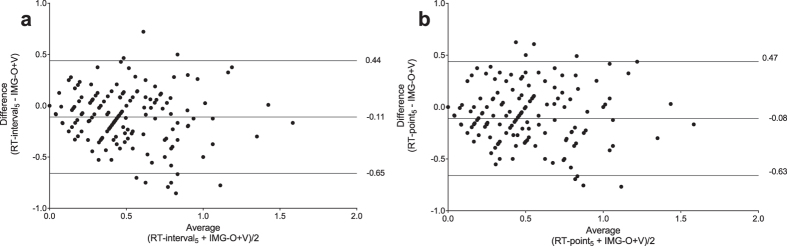
Bland and Altman plots comparing image and real-time scores. and RT-interval_5_ or RT-point_5_. The Bland-Altman analysis indicates that the limits of agreement between (**A**) Real-time interval observation over 5 minutes (RT-interval_5_) with a bias (underestimation) by real-time scores of −0.11 and limits of agreement ranging from −0.65 to 0.44. (**B**) Real-time point observation over 5 minutes (RT-point_5_) with a bias (underestimation) by real-time scores of −0.08 and limits of agreement ranging from −0.63 to 0.50.

The Bland and Altman analysis revealed that the bias between real-time and standard RGS observation methods was small, regardless of the type or frequency of real-time observations, and represented a systematic underestimation of the standard method by real-time methods of approximately 0.1 (Table 1). The limits of agreement (bias ± 2 SD) reflect the distribution of 95% of the measured differences between scoring methods. Observation frequencies of either 5 or 10 minutes showed similar limits of agreement for both interval and point observations (Table 1, Fig. 4). As observation frequency decreased to 2 minutes, the limits of agreement widened (Table 1, Fig. S1).

**Figure 5:**
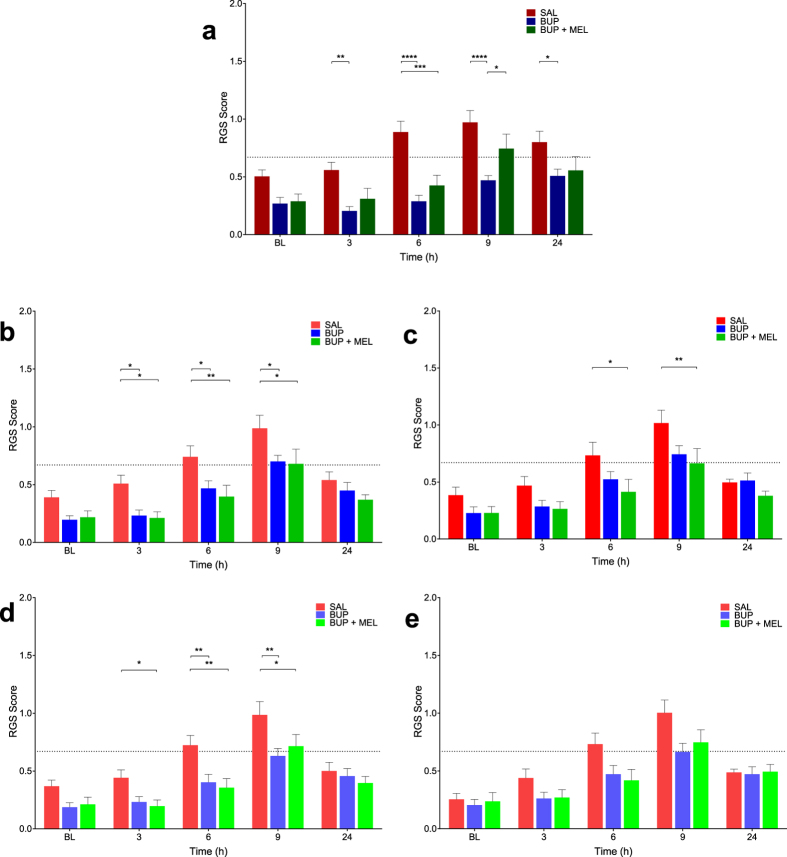
Both standard Rat Grimace Scale (RGS) and real-time interval RGS scoring were able to discriminate between saline and analgesia treatment groups. (**A**) Standard (video-based) RGS scoring (IMG-O + V). Lower RGS scores were observed in the buprenorphine treatment group at 3 (p = 0.007), 6 (p < 0.0001), 9 (p < 0.0001) and 24 h (p = 0.03). RGS scores were reduced in the multimodal treatment group at 6 h (p = 0.0003) and a difference was observed between buprenorphine and multimodal treatment groups at 9 h (p = 0.04). (**B**) Real-time interval observation over 10 minutes (RT-interval_10_). RGS scores were lower in the buprenorphine group at 3 (p = 0.03), 6 (p = 0.03), and 9 h (p = 0.02). Similarly, multimodal analgesia (buprenorphine and meloxicam) resulted in a decrease in RGS scores at 3 (p = 0.02), 6 (p = 0.004) and 9 h (p = 0.01). (**C**) The real-time point observation over 10 minutes (RT-point_10_) identified a treatment effect in the multimodal treatment group at 6 h (p = 0.02) and 9 h (p = 0.007). (**D**) Real-time interval observation over 5 minutes (RT-interval_5_) showed that buprenorphine and multimodal analgesia were associated with a decrease in RGS scores at 6 h (buprenorphine, p = 0.005; multimodal, p = 0.001) and 9 h (buprenorphine, p = 0.002; multimodal, p = 0.02). RGS scores were also lower in the multimodal group at 3 hours (p = 0.04). (**E**) Real-time point observation over 5 minutes (RT-point_5_) did not identify analgesia treatment effects (p = 0.08). SAL = saline, BUP = buprenorphine, MEL = meloxicam. Data are mean ± SEM. Broken horizontal line represents a previously derived analgesic intervention threshold^16^.

Most (4/6) of the real-time observation methods, including all of the interval observation methods, were able to discriminate between saline and analgesic treatments (Fig. 5, S2). Buprenorphine and the multimodal treatments provided effective analgesia with significant reductions in RGS scores. Coinciding with an expected peak in carrageenan-induced pain at 6 hours^13^, buprenorphine and multimodal analgesia were effective at reducing RGS scores compared with saline in the IMG-O+V (buprenorphine, p < 0.0001, 95% CI: 0.33 to 0.87; multimodal, p = 0.0003, 95% CI: 0.19 to 0.74, Fig. 5a), RT-interval_10_ (buprenorphine, p = 0.03, 95% CI: 0.02 to 0.52; multimodal, p = 0.004, 95% CI: 0.09 to 0.60, Fig. 5b), RT-point_10_ (multimodal, p = 0.02, 95% CI: 0.05 to 0.59, Fig. 5c), RT-interval_5_ (buprenorphine, p = 0.005, 95% CI: 0.08 to 0.56; multimodal, p = 0.001, 95% CI: 0.13 to 0.61, Fig. 5d). The same pattern was observed at 9 hours in the RT-interval_10_ (buprenorphine, p = 0.02, 95% CI: 0.03 to 0.54, multimodal, p = 0.01, 95% CI: 0.06 to 0.56, Fig. 5b), RT-point_10_ (multimodal, p = 0.007, 95% CI: 0.08 to 0.62, Fig. 5c) and RT-interval_5_ (buprenorphine, p = 0.002, 95% CI: 0.12 to 0.60, multimodal, p = 0.02, 95% CI: 0.03 to 0.51, Fig. 5d). At 9 hours the IMG-O+V method identified a decrease in RGS scores associated with buprenorphine compared with saline (p < 0.0001, 95% CI: 0.23 to 0.78) and multimodal analgesia (p = 0.04, 95% CI: 0.01, 0.54, Fig. 5a). Fewer differences were observed at 3 and 24 hours, consistent with the expected time course of carrageenan-induced inflammation. No analgesic effects were identified with RT-point_5_ (F = 2.73, df 2, p = 0.08, Fig. 5e). Ability to discriminate between saline and analgesic treatment groups were identifiable with RT-interval_2_ but not RT-point_2_ (Fig. S2).

When comparing the RT-point observations with IMG-O+V, the expected pattern of RGS scores with different treatments is present (Fig. S3). *Single interval and point observation scoring methods.*

**Figure 6:**
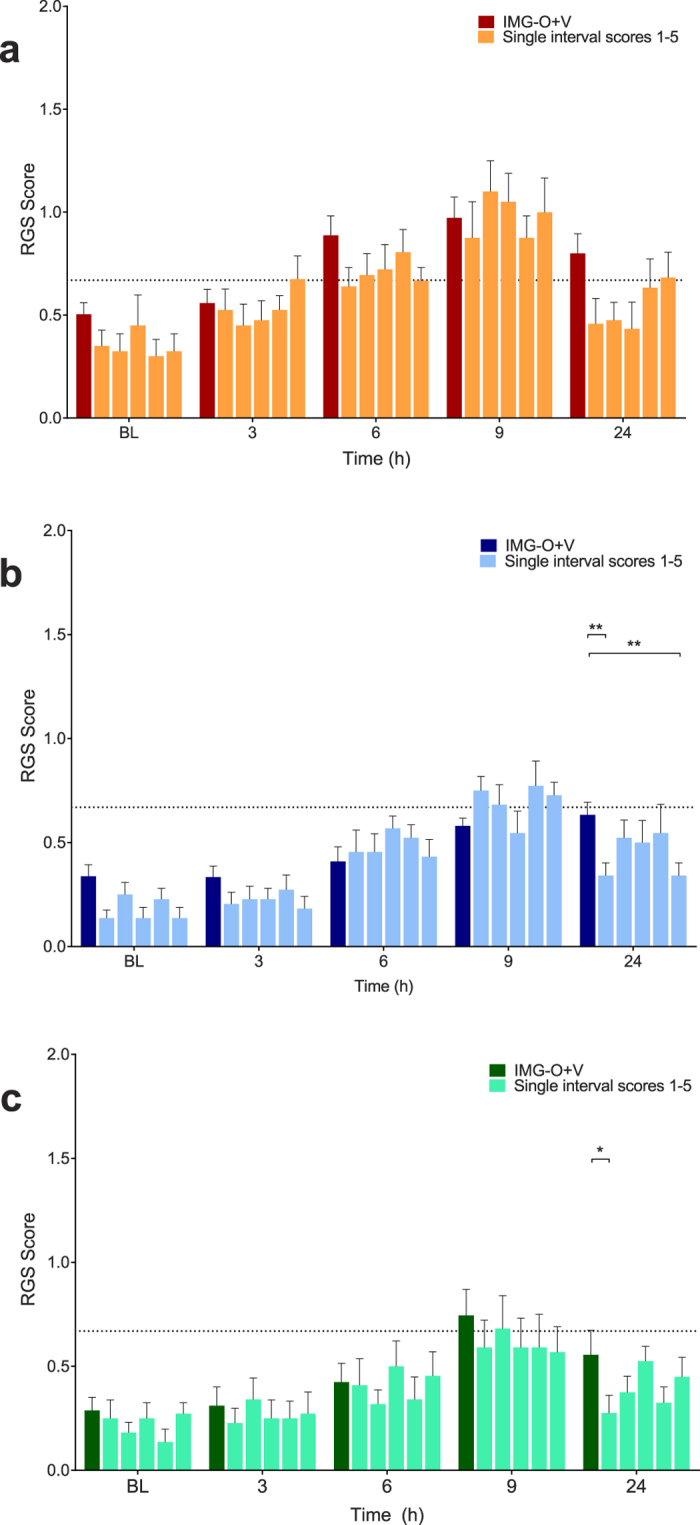
Single real-time interval scores (scores 1–5) approximates the expected time course associated with each treatment, but visual inspection of the data reveals substantial variability between scores. (**A**) Saline treatment group. There was no main effect of treatment (p = 0.11). (**B**) Buprenorphine treatment group. A significant difference between scores was observed at 24 hours (p = 0.003). (**C**) Multimodal treatment group. A significant difference was observed at 24 hours (p = 0.03). Data are mean ± SEM. Broken horizontal line represents a previously derived analgesic intervention threshold^16^.

**Figure 7:**
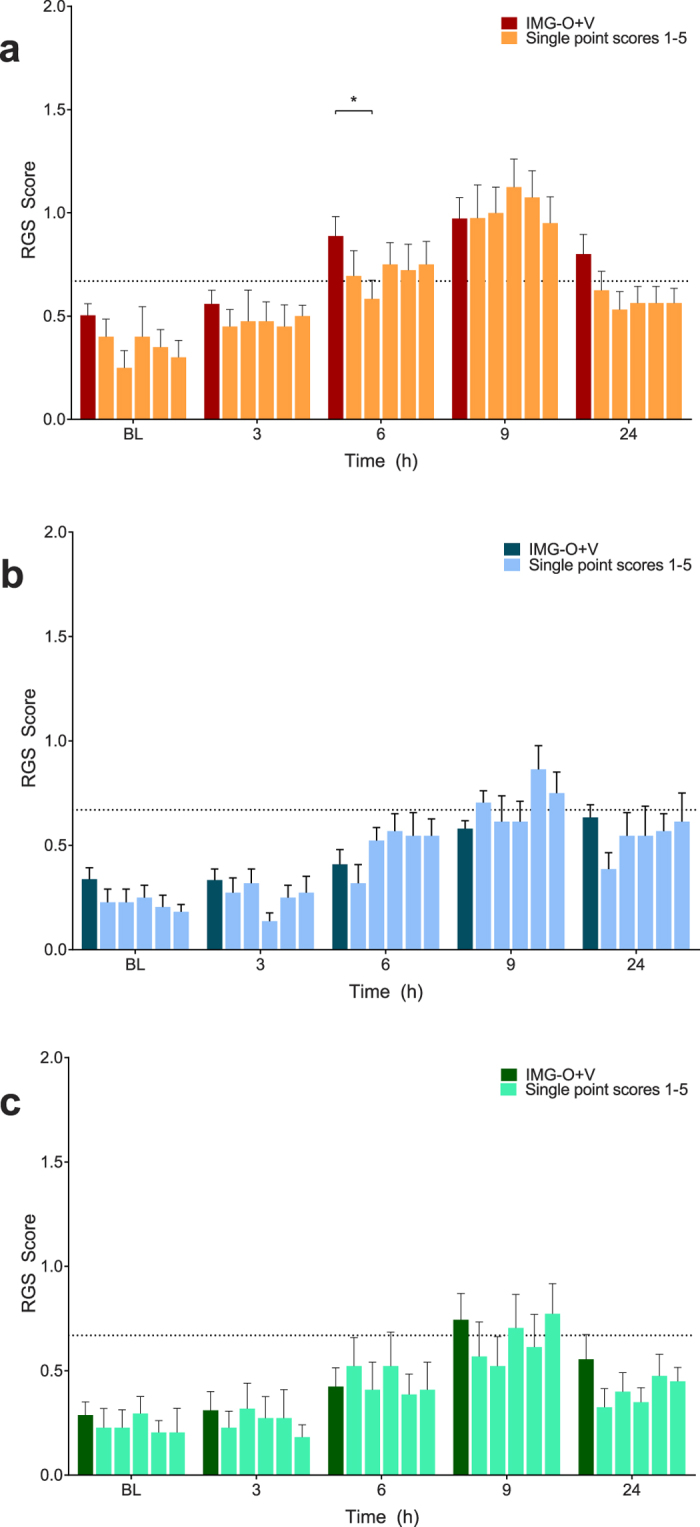
Single real-time point scores (scores 1–5) approximates the expected time course associated with each treatment, but visual inspection of the data reveals substantial variability between scores. No main effects for scoring method were identified in the buprenorphine ((**B**) p = 0.13) and multimodal ((**C**) p = 0.16) treatment groups. A single difference was observed at 6 hours in the saline group ((**A**) p = 0.03). Data are mean ± SEM. Broken horizontal line represents a previously derived analgesic intervention threshold^16^.

The random selection of 5 interval and 5 point observations illustrated that the predicted time course of pain for each treatment group was present but substantial variability was observed between individual scores (Figs 6 and 7).

### Observer effect

**Figure 8:**
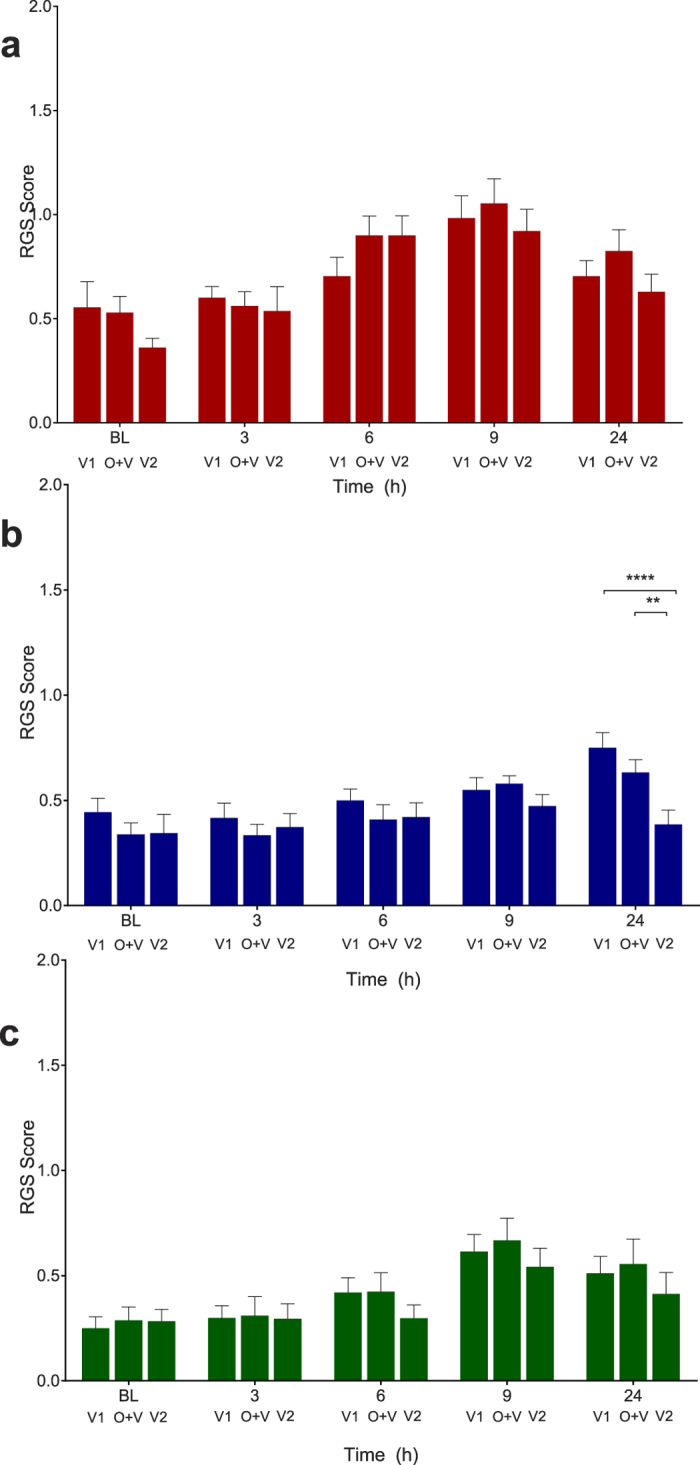
Presence of the observer had a minimal effect on Rat Grimace Scale (RGS) scores. No observer effect was observed in the saline (**A**) p = 0.30) and multimodal treatment groups (**C**) p = 0.28). A significant difference between observation periods was present in the buprenorphine group (**B**) at 24 hours, between V1 and V2 (p < 0.0001) and between IMG-O+V and V2 (p = 0.01). V1 and V2 = video only, no observer present. O+V = video, with observer present. Data are mean ± SEM. Broken horizontal line represents a previously derived analgesic intervention threshold^16^.

The presence of the observer did not significantly affect the RGS scores from the saline (F = 1.27, df 2, p = 0.30; Fig. 8a) and multimodal analgesia treatment groups (F = 1.37, df 2, p = 0.28, Fig. 8c). Unexpectedly, significant differences were observed at 24 h in the buprenorphine group between observation periods V1 and V2 (p < 0.0001, 95% CI: 0.17 to 0.56) and between IMG-O+V and V2 (p = 0.01, 95% CI: 0.05 to 0.44, Fig. 8b).

### Control groups

None of the control treatments resulted in significant changes to RGS scores compared with baseline values (Table S1).

### Pica

There was no evidence of pica behaviour from necropsy examination or masses of petri dishes in the treatment groups (Table S2). The buprenorphine control groups exhibited a small amount of pica behaviour (petri dish weight changes of 0.1–0.6 g, Table S3).

## Discussion

The appeal of real-time application of rodent grimace scales lies in expanding their current role as retrospective research instruments to one allowing early identification of pain, facilitating timely intervention and improving the welfare of laboratory rodents. The potential for rodent grimace scales to be applied as a real-time scoring system has been previously suggested^11^,^16^,^20^ and attempted with limited success in mice^17^,^21^.

We have shown that real-time RGS scoring is an accurate and feasible alternative to the standard method described by Sotocinal *et al*.^12^, offering a refinement to the humane care of laboratory rats. The ability of a new method to reflect changes identified by the current (criterion) standard shows accuracy and construct validity. In evaluating different methods of real-time scoring we identified multiple 15 s interval observations as more sensitive than multiple point observations. And we observed that single observations, both interval and point, approximated the predicted time course of pain, but exhibited substantial variability. Applying the Bland and Altman method to our data allowed assessment of systematic differences between observation methods and the variability around these differences. There was a small systematic underestimation by all the real-time methods, showing that on average, real-time scores are very close to image-generated scores. The similarity between 5 and 10-minute real-time observation periods indicates that 10-minute observation periods are unnecessary if the RGS is being applied as a tool to guide pain management (rather than as a research tool). Furthermore, the similarity between RT-interval_5_ and RT-point_5_ observations offers alternative means of scoring depending on user preference. The acceptability of a new (real-time) technique over a criterion standard (image-based) depends on a subjective assessment of the limits of agreement. For RT-interval_5_ and RT-point_5_ observations, the limits of agreement span a 0.5 score range either side of the bias. Therefore, there is the possibility of a single observation either over or underestimating the true score. Furthermore, the Bland and Altman plots show that data variability increases at RGS scores >0.5. Interpreting these observations together, a practical approach could be a planned reassessment of any animal with an initial RGS score >0.5 within a relatively short period (e.g. 1 hour), taking in to account the potential for suffering if providing analgesia is delayed against any side-effects associated with analgesic use. As RGS scores exceed a previously identified threshold for intervention (RGS score >0.67)^16^, the likelihood of an animal experiencing pain increases, in which case the reassessment interval should be kept short or analgesia provided immediately and the animal reassessed for an improvement in RGS score.

The agreement between RT scores and IMG scores was not reflected in their ability to discriminate treatment effects statistically as observations decreased to 2 minutes. Both interval and point observation methods (RT-interval_10_ and RT-point_10_) were able to discriminate between the saline and analgesic treatments at the 6 and 9 hour time points, when peak RGS scores are expected^13^,^22^ and did not differ significantly from the standard RGS scoring method. Furthermore, the mean scores at these times exceeded a proposed analgesic intervention threshold^16^, providing evidence for the relevance of this decision-making tool. However, when the observation period was decreased to 5- or 2-minutes (RT-interval_5,2_ and RT-point_5,2_) only the interval scoring methods were able to reliably discriminate between saline and analgesia treatment groups, though the pattern of RGS scores did exhibit the expected time courses of the different treatment groups. This inability to discriminate was likely due to insufficient power when scoring with RT-point_5,2_ as the Bland and Altman results showed similar agreement to the equivalent interval scoring methods.

Our findings agree with those of Ballantyne *et al*.^23^, where a multidimensional 7 item pain scale, of which 3 items were facial action units, was evaluated in neonatal infants during painful and non-painful procedures^23^. The authors showed that real-time (bedside) observations (over a 45 s period) did not differ significantly from the standard video-based assessments and were able to discriminate between predicted painful and non-painful states. This assessment method is similar to the successful interval method we employed.

Faller *et al*.^21^ successfully used the mode of observed scores (scored from 10 photographs taken over a 15–20 minute observation period) to identify a reduction in the MGS score following buprenorphine administration^21^. This approach resembles our point observations, though the discriminatory ability identified differs from our findings with the RT-point_10_ observation method, where 18 observations were recorded over a 10 minute period. However, a direct comparison between studies is limited by differences in the time allowed to perform the scoring (photograph *versus* live observation), species and grimace scales (the number of facial action units differs between the RGS and MGS).

The similarity in RGS scores we observed between RT-interval and standard RGS methods differs from the findings of Miller and Leach (2015)^17^ where they reported, using the MGS, that real-time scores were significantly lower than image scores in 6/7 comparisons (across strain and gender). Their real-time scoring was based on 3 × 5 s observations during a 10 minute observation period and image scores were derived from 3 randomly selected photographs taken during the same 10 minute period. Our RT-interval_2_ and RT-point_2_ observations at baseline provide the closest comparison to this study as the mice studied did not receive potentially painful interventions. While our results showed no significant differences between these observation types and the standard RGS method, only interval observations were capable of differentiating treatment effects. As suggested by the authors, the use of photographs to generate MGS scores may have resulted in an artificial elevation of scores by capturing behaviours interfering with scoring (such as blinking). A comparison with the standard RGS scoring method^11^ would allow evaluation of this possibility. Single observations with both the RT-interval and RT-point methods displayed the predicted time course for each treatment group, with RGS scores in the saline group exceeding a proposed threshold for analgesic intervention at 9 hours, in contrast to the buprenorphine and multimodal groups^16^. However, visual inspection of the data revealed substantial variability with both observation methods, indicating that reliance on a single observation for treatment decisions is insufficient, with the risk of failing to identify a painful state.

Buprenorphine was an effective analgesic, limiting the predicted increase in RGS scores at 6 and 9 hours after carrageenan administration^13^,^22^. The timing of buprenorphine administration may have resulted in its analgesic effects waning around the 9 hour time point^24^, explaining the slight increases in RGS scores observed at this time in the buprenorphine and multimodal groups. The optimal dosing interval for buprenorphine in rats is unclear and is likely to vary according to procedure and strain, highlighting the importance of regular pain assessment with an appropriate instrument^18^,^24^,^25^. The choice of a 0.03 mg/kg dose was based on recent work showing its efficacy when evaluated with the RGS^9^. A dose of 0.05 mg/kg may have provided a longer duration of analgesia^24^ but has been associated with pica behaviour^18^,^26^. Therefore, the lower dose was selected to minimise the possibility of pain from pica behaviour acting as a confounding factor.

Somewhat unexpectedly, the multimodal treatment group (buprenorphine and meloxicam) exhibited similar RGS scores to the buprenorphine treatment group at all time points, when it might be expected that a multimodal analgesic approach with a non-steroidal anti-inflammatory agent (NSAID) and opioid would result in lower RGS scores^3^,^27^,^28^. There are several interpretations of these findings. Firstly, the addition of meloxicam may not have conferred any additional benefit as the RGS scores were already low and below a level identified as painful^16^. Secondly, the relationship between inflammation and pain may be less clear than previously believed. Meloxicam may reduce inflammation without a concurrent decrease in pain^20^,^29^. However, this contradicts a substantial body of evidence that NSAIDs are effective analgesics in rats^24^,^30^,^31^,^32^, though the relationship between the behavioural (postural) pain scale used in those studies and the RGS is undefined. Finally, the RGS may not be sensitive enough to identify subtle variations in pain levels. This is possible as original work validating the RGS used the potent opioid morphine to demonstrate analgesic sensitivity (construct validity) in several robust pain models^12^.

RGS scores were similar between observation periods (V1, O+V, V2), indicating that the presence of an observer had negligible impact. The extent to which this lack of effect was related to the observer being female is unknown: a systematic effect of observer gender has been recently shown in mice, with a reduction in MGS scores in the presence of men as a result of stress-induced analgesia^33^. The exception to the general case was the difference observed between observation periods at 24 hours in the buprenorphine group. This is unlikely to be an ‘observer effect’ as this difference was limited to a single treatment group and time point. Furthermore, if an observer effect was present, RGS scores from V1 and V2 periods would be expected to be similar, and different from those generated during O + V.

Scoring by an observer involved with the study raised the possibility of observer bias as it was not possible to blind to time point. This may have affected the real-time RGS scores at baseline and 24 hours, when RGS scores would be predicted to be low for this model. This possibility was addressed by comparing real-time scores with those generated from randomised, blinded images. Without concurrent video-recording, observer bias cannot be accounted for unless the observer has no knowledge of the study design. This may reflect the situation encountered if real-time RGS scoring were to be used by technicians or veterinarians not involved with a study.

We have shown that the RGS can be successfully applied with real-time observations, lending itself to use as a rapid pain assessment tool to identify acute pain in rats. Interval observations over a 2 minute period were able to discriminate between treatment effects whereas point observations displayed lower sensitivity and were unable to discriminate between treatments. Single observations, interval or point, showed substantial variability and should not be used to determine analgesic administration without planned reassessment. The best balance between practicality and accuracy is achieved with 5-minute observation periods with either interval or point observations. When using real-time observations, we suggest implementing planned reassessments to account for score variability, particularly as RGS scores exceed 0.5. However, the decision to administer analgesia should be balanced against the welfare cost of delaying intervention for reassessment.

## Additional Information

**How to cite this article**: Leung, V. *et al*. Real-time application of the Rat Grimace Scale as a welfare refinement in laboratory rats. *Sci. Rep.* **6**, 31667; doi: 10.1038/srep31667 (2016).

## Supplementary Material

Supplementary Information

## Figures and Tables

**Table 1:** Bland and Altman method comparing each real-time (RT) observation method with image (IMG) scores.

Observation type	Bias	Upper limit	Lower limit
RT-interval_10_	−0.09	0.46	−0.63
RT-interval_5_	−0.11	0.44	−0.65
RT-interval_2_	−0.14	0.43	−0.71
RT-point_10_	−0.07	0.49	−0.63
RT-point_5_	−0.08	0.47	−0.63
RT-point_2_	−0.09	0.50	−0.68

Bias is the mean difference between RT and IMG Rat Grimace Scale scores. Upper and lower limits of agreement are mean difference ± 2 SD.
