# Supplementary material for: Real-time application of the Rat Grimace Scale as a welfare refinement in laboratory rats
Source: Sci Rep. 2016 Aug 17;6:31667. doi: 10.1038/srep31667 (PMC4987703; doi:10.1038/srep31667)
Supplement: Supplementary Information [file srep31667-s1.pdf]

Real-time application of the Rat Grimace Scale as a welfare refinement in laboratory rats

Vivian Leung, Emily Zhang, Daniel SJ Pang

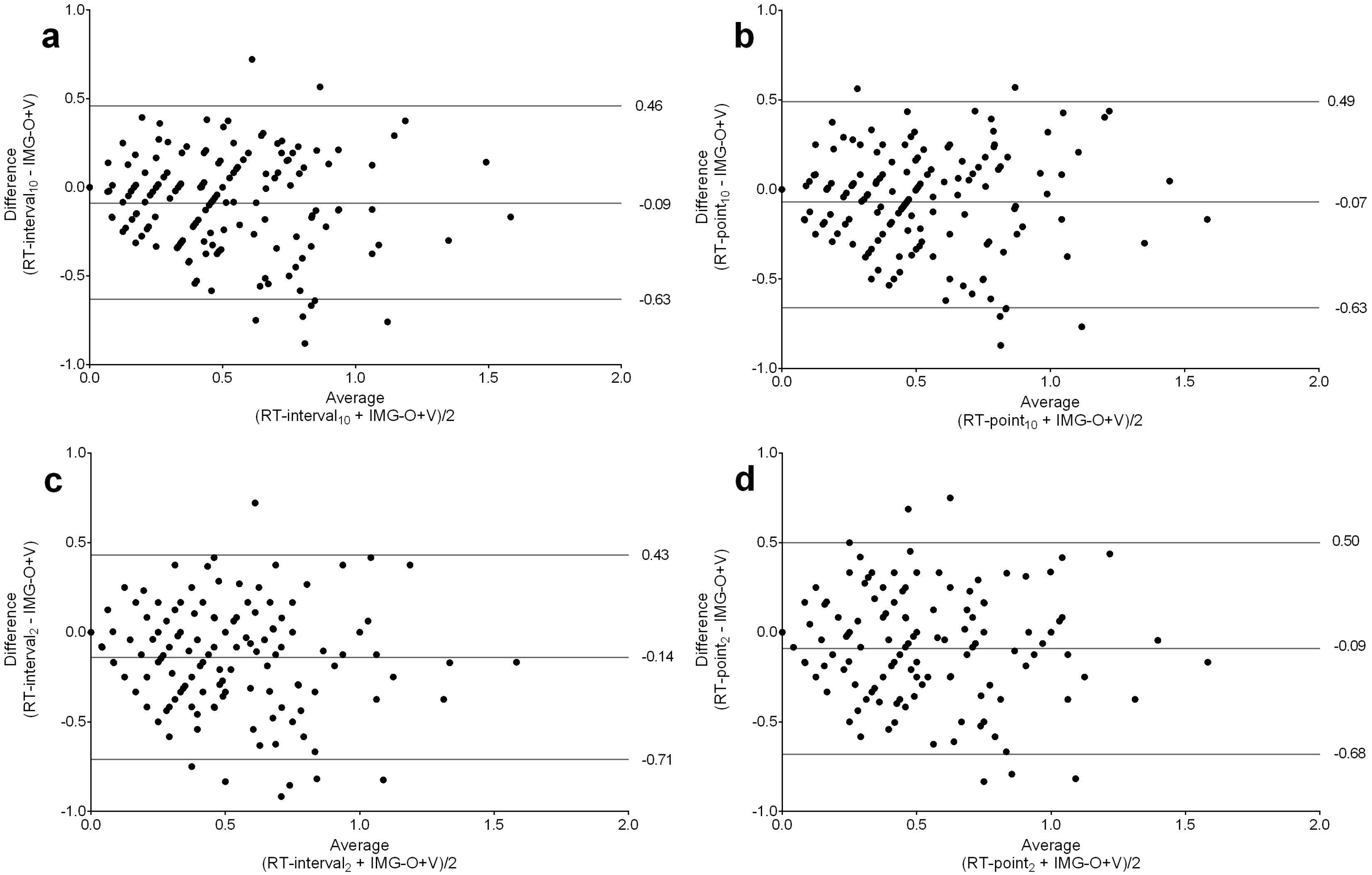

Figure S1: Bland and Altman plots comparing real-time (RT) scoring (RT-interval$_{10,2}$ and RT-point$_{10,2}$) to image-based (IMG) scores for (a) RT-interval$_{10}$, (b) RT-point$_{10}$, (c) RT-interval$_{2}$, (d) RT-point$_{2}$. Data are mean difference (bias, central horizontal line) and limits of agreement (bias ± 2 SD, upper and lower horizontal lines).

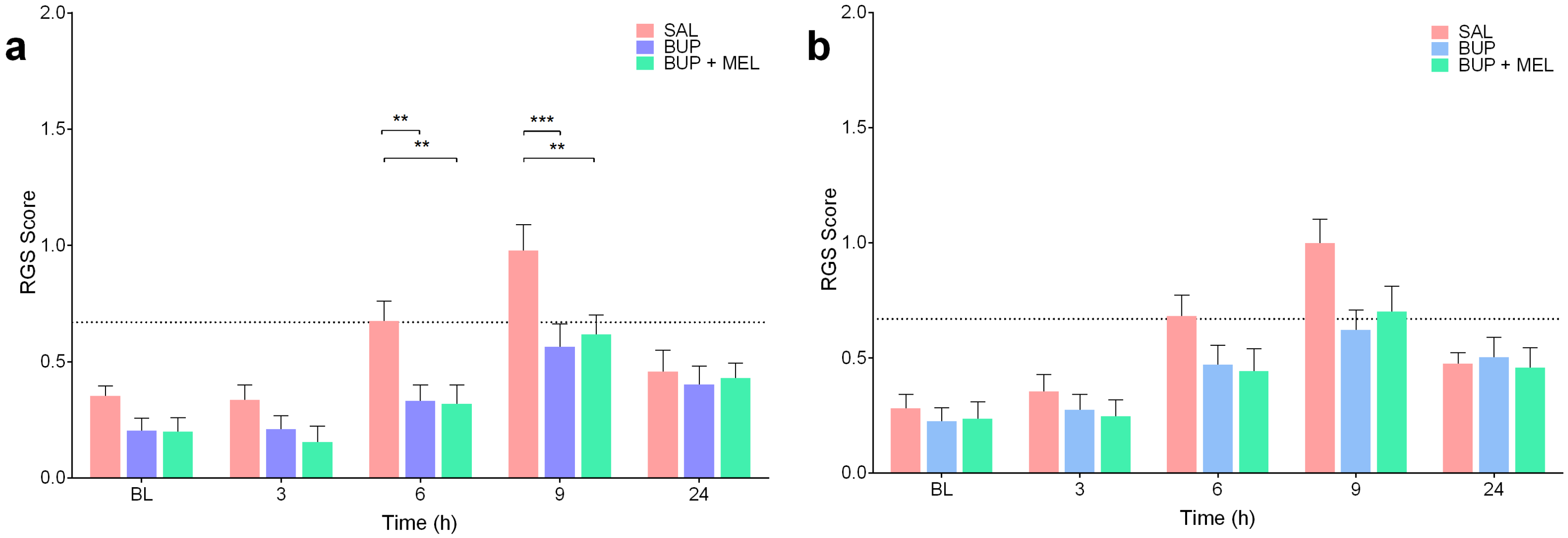

Figure S2: Treatment effects were identifiable with RT-interval$_2$ but not RT-point$_2$. (a) The real-time interval observation over 2 minutes (RT-interval$_2$) identified treatment effect in the buprenorphine and multimodal treatment groups from saline treatment group at 6h (buprenorphine, p = 0.005, 95% CI: 0.09 to 0.60; multimodal, p = 0.003, 95% CI: 0.10 to 0.61) and 9h (buprenorphine, p = 0.0005, 95% CI: 0.16 to 0.67; multimodal, p = 0.003, 95% CI: 0.11 to 0.62). (b) Real-time point observation over 2 minutes (RT-point$_2$) was not able to discriminate between analgesia and saline treatment groups (p = 0.19). SAL = saline, BUP = buprenorphine, MEL = meloxicam. Data are mean ± SEM. Broken horizontal line represents a previously derived analgesic intervention threshold (Oliver et al., 2014).

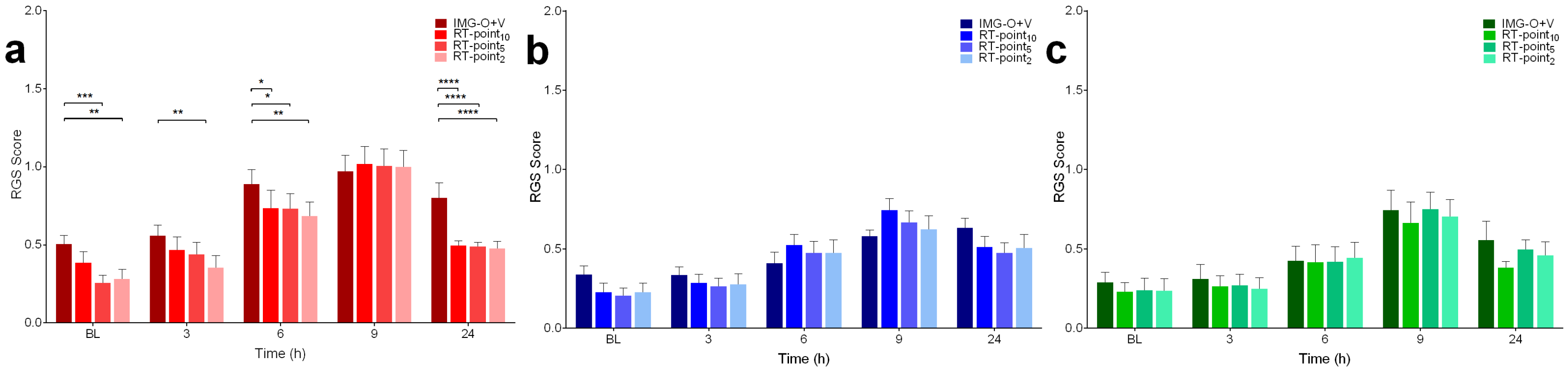

Figure S3: **Real-time (RT) point scoring methods compared to standard (video-based) Rat Grimace Scale (RGS) scoring (IMG-O+V)**. (a) Scoring method was found to differ significantly in the saline treatment groups: IMG-O+V *vs.* RT-point$_{10}$ at 6 ($p = 0.04$, 95% CI: 0.01 to 0.31) and 24h ($p < 0.0001$; 95% CI: 0.15 to 0.45), IMG-O+V *vs.* RT-point$_5$ at BL ($p = 0.0004$; 95% CI: 0.10 to 0.37), 6 ($p = 0.04$; 95%CI: 0.01 to 0.31) and 24h ($p < 0.0001$; 95% CI: 0.16 to 0.46), IMG-O+V *vs.* RT-point$_2$ at BL ($p = 0.002$; 95% CI: 0.07 to 0.37), 3 ($p = 0.005$; 95% CI: 0.05 to 0.36), 6 ($p = 0.005$; 95% CI: 0.05 to 0.36) and 24h ($p < 0.0001$; 95% CI: 0.17 to 0.47). There was no effect of scoring method on RGS scores in (b) buprenorphine ($F = 1.38$, df 3, $p = 0.27$) and (c) multimodal treatment groups ($F = 2.89$, df 3, $p = 0.05$). BL = baseline. Data are mean ± SEM.

	Time point								
Treatment	BL Median (IQR)	3 Median (IQR)	p-value	6 Median (IQR)	p-value	9 Median (IQR)	p-value	24 Median (IQR)	p-value
A	0.40 (0.34, 0.48)	0.38 (0.33, 0.67)	>0.99	0.63 (0.44, 0.75)	0.47	0.36 (0.27, 0.41)	>0.99	0.40 (0.22, 0.67)	>0.99
B	0.13 (0.08, 0.17)	0.25 (0.19, 0.31)	0.88	0.31 (0.19, 0.47)	0.29	0.28 (0.06, 0.36)	0.88	0.57 (0.31, 0.91)	0.06
C	0.17 (0.04, 0.33)	0.50 (0.23, 0.71)	>0.99	0.42 (0.25, 0.58)	0.23	0.54 (0.31, 0.83)	0.10	0.63 (0.13, 0.75)	0.58
D	0.25 (0.19, 0.31)	0.29 (0.19, 0.83)	>0.99	0.21 (0.10, 0.44)	>0.99	0.46 (0.25, 0.82)	0.72	0.31 (0.25, 0.66)	0.58
E	0.29 (0.02, 0.50)	0.50 (0.20, 0.65)	0.71	0.50 (0.23, 0.65)	0.47	0.42 (0.23, 0.57)	>0.99	0.31 (0.25, 0.53)	>0.99
F	0.19 (0.13, 0.31)	0.50 (0.25, 0.63)	0.29	0.46 (0.10, 0.81)	0.72	0.33 (0.27, 0.46)	>0.99	0.38 (0.25, 0.78)	0.23

Supplementary Table S1: Rat Grimace Scale scores from control group. Scores generated by standard (video-based) method. Scores at 3, 6, 9 and 24h were compared to their BL scores. A: saline (buprenorphine volume) + saline (meloxicam volume) + anesthesia + intra-plantar saline; B: buprenorphine + anesthesia + intra-plantar saline; C: buprenorphine + meloxicam + anesthesia + intra-plantar saline; D: buprenorphine + anesthesia; E: buprenorphine and F: buprenorphine and meloxicam. There were no significant differences in RGS scores at the various time points compared to their baseline scores. BL = baseline. IQR = interquartile range.

Treatment group	Cage #	Petri dish weight (g)		
		before (BL)	after	difference
Saline	1	8.6	8.6	0
	2	2.7	2.7	0
	3	6.9	6.9	0
	4	8.5	8.5	0
	5	8.4	8.4	0
	6	7.2	7.2	0
Buprenorphine	1	8.6	8.6	0
	2	6.7	6.7	0
	3	-	-	0
	4	8.2	8.2	0
	5	8.5	8.4	0.1
	6	8.2	8.2	0
Buprenorphine + Meloxicam	1	8.6	8.6	0
	2	8.0	8.0	0
	3	8.7	8.7	0
	4	8.6	8.6	0
	5	6.0	6.0	0
	6	7.8	7.8	0

Supplementary Table S2: **Weights of petri dishes in the three treatment groups.** No non-food items were found in the stomach of all the rats and the weights of petri dishes did not change from their baseline weights. Variation in weights of petri dishes “before” reflects chewing which occurred during habituation. BL = baseline. Difference = before (BL) – after.

Treatment group	Cage #	Crossover #	Petri dish weights (g)		
			before (BL)	after	difference
A	1	1	7.8	7.8	0
	3	2	7.7	7.7	0
B	2	2	6.3	6	0.3
	4	2	8.1	-	-
C	3	1	7.7	7.7	0
	1	3	5.5	5.5	0
D	2	1	7.9	7.9	0
	4	1	7.9	7.9	0
E	2	3	5.1	4.5	0.6
	4	3	4.2	3.8	0.4
F	1	2	8	7.8	0.2
	3	3	6.3	6.2	0.1

Supplementary Table S3: **Weights of petri dishes in the control groups. (**A) saline (buprenorphine volume) + saline (meloxicam volume) + anesthesia + intraplantar saline. (B) buprenorphine + anesthesia + intraplantar saline (C) buprenorphine + meloxicam + anesthesia + intraplantar saline (D) buprenorphine + anesthesia (E) buprenorphine and (F) buprenorphine and meloxicam. No non-food items were found in the stomach of all the rats and unlike the rats given intra-plantar carrageenan (Table S2), weights of petri dishes did decrease from their baseline weights when rats were given buprenorphine. Variation in weights of petri dishes “before” reflects chewing which occurred during habituation. BL = baseline. Difference = before (BL) – after.
